# Biomarkers of Tuberculous Meningitis and Pediatric Human Immunodeficiency Virus on the African Continent

**DOI:** 10.3389/fneur.2022.793080

**Published:** 2022-05-19

**Authors:** Charlotte Elisabeth Teunissen, Ursula Rohlwink, Dasja Pajkrt, Petrus J. W. Naudé

**Affiliations:** ^1^Neurochemistry Laboratory, Department of Clinical Chemistry, Amsterdam Neuroscience, Amsterdam University Medical Centers, Vrije Universiteit, Amsterdam, Netherlands; ^2^Division of Neurosurgery, Neuroscience Institute, Department of Surgery, University of Cape Town, Cape Town, South Africa; ^3^The Francis Crick Institute, London, United Kingdom; ^4^Department of Pediatric Infectious Diseases, Amsterdam University Medical Centers, Location Academic Medical Center, Amsterdam, Netherlands; ^5^Department of Psychiatry and Mental Health, Neuroscience Institute, University of Cape Town, Cape Town, South Africa

**Keywords:** biomarkers, tuberculous meningitis, HIV, inflammation, cerebrospinal fluid, blood plasma/serum

## Abstract

Biomarkers in body fluids are helpful objective tools in diagnosis, prognosis and monitoring of (therapeutic) responses of many neurological diseases. Cerebrospinal fluid (CSF) biomarkers are part of the diagnostic toolbox for infectious neurological diseases. Tuberculous meningitis (TBM) and Human immunodeficiency virus (HIV), are important burdens of disease in Africa and can negatively affect brain health. Two thirds of the world's population of people living with HIV reside in sub-Saharan Africa and 25% of the global burden of tuberculosis (TB) is carried by the African continent. Neuroinflammation and damage of specific neuronal cell types are key constituents in the pathophysiology of these central nervous system (CNS) diseases, and important potential sources of circulating biomarkers. In this review, we summarize current research in the use of biomarkers in TBM and pediatric HIV as case demonstrations for high prevalence neurological diseases in Africa. Inflammatory molecules, primarily when detected in CSF, appear to have diagnostic value in these diseases, especially when measured as profiles. Brain injury molecules, such as S100, Neuron specific enolase and glial fibrillary acidic protein may have prognostic value in TBM, but more studies are needed. There is a need for more cost-economic and high sensitivity technologies to drive further biomarker discoveries and translate into healthcare improvements for these important healthcare problems in a globally fair way.

## Introduction

Biomarkers in body fluids are helpful objective tools in diagnosis, prognosis and monitoring of (therapeutic) responses of many neurological diseases. Cerebrospinal fluid (CSF) biomarkers are part of the diagnostic toolbox for chronic neurological diseases such as Alzheimer's disease and Multiple Sclerosis, and for infectious central nervous system (CNS) diseases such as meningitis (1, 2). Biomarkers previously tested exclusively in the CSF compartment in neurological diseases, can nowadays be measured in the systemic blood as well, and blood and CSF neurobiomarkers are progressively being used as useful endpoint measurements in trials targeting CNS diseases (3, 4). Acute and chronic infections, including tuberculous meningitis (TBM) and Human immunodeficiency virus (HIV), are important burdens of disease in Africa and can have detrimental effects on brain health. Two thirds of the world's population of people living with HIV reside in sub-Saharan Africa and 25% of the global burden of tuberculosis (TB) is carried by the African continent (5, 6). Neuroinflammation and damage of specific neuronal cell types are key constituents in the pathophysiology of these CNS diseases, and important potential sources of circulating biomarkers. Although biomarker research for infectious CNS diseases is not as intensively studied as for some other neurological conditions, there are interesting pilot data from which parallels with widely studied disorders can be drawn, and which highlight the need for further research into the diagnostic and prognostic potential of biomarkers in the African context. In this review, we will summarize current research on biomarkers in TBM and pediatric HIV as case demonstrations for high prevalence neurological disease in Africa and discuss options for biomarker development with consideration for the unique challenges on the continent.

## Tuberculous Meningitis

In the World Health Organisation's 2020 TB Report TB was remained the deadliest infectious disease globally. In 2019 the international TB incidence was 10 million, with numerous countries within Africa ranking amongst those carrying the largest global burden of TB (6). TBM is estimated to occur in one out of 100 TB cases (7), and is the most fatal and debilitating manifestation of TB, leading to high rates of death and disability in adults and children (8).

### Pathogenesis of TBM

Tuberculosis infection occurs after the inhalation of infectious droplets of aerosolised *Mycobacterium tuberculosis* (*Mtb*), which stimulates an innate immune response in the lung tissue that leads to the containment of the bacilli within a granuloma (9). However, in the elderly, immune compromised or very young, the infection may progress to active TB disease associated with destruction of the lung parenchyma and dissemination of the TB bacillus to other organ systems, including the CNS (10). Despite the protective blood brain barrier (BBB), *Mtb* gains access to the brain through numerous postulated mechanisms, including rearrangement of the actin cytoskeleton of brain microvascular endothelial cells (11), bacillary endothelial adhesion (12), or the Trojan Horse whereby *Mtb* is trafficked into the CNS in infected innate immune cells (13). The limited resident CNS immunity facilitates bacillary survival and replication and the development of silent tuberculous lesions, often referred to as the Rich's Focus, which be located on the cortical pia or adjacent to the ventricles and meninges (14). Rupture of these lesions is thought to result in granulomatous inflammation.

*Mycobacterium tuberculosis* is recognized by the brain's resident immune cells, microglia, through pattern recognition receptors including toll-like receptors. Activation of microglia leads to secretion of a number of pro-inflammatory mediators (discussed below), recruitment of peripheral immune cells and activation of astrocytes which aid in the immune response (15). The cerebral immune response is an important determinant of poor outcome as the formation of thick inflammatory exudate causes cerebral vasculitis and occlusion as well as hydrocephalus and raised intracranial pressure. Consequently, the brain is at high risk of ischaemia and infarcts are seen in almost 70% of patients (16).

A delay in starting treatment is a major determinant of poor outcome, yet timely diagnosis of TBM is challenging due to its non-specific presentation (17). Similarly, clinical tools are limited in accurately predicting patient outcomes making it difficult to triage limited resources to patients at greatest risk. Biomarker studies have, therefore, aimed to identify markers to improve accurate and early diagnosis and prognosis. Biomarkers may also serve as valuable proxy measures of novel treatment efficacy, and to elucidate disease pathophysiology and new intervention strategies.

### Inflammatory Biomarkers

Numerous cytokines and chemokines are elevated in the CSF of adult and pediatric TBM patients, including tumor necrosis factor (TNF)-α, interferon (IFN)-γ, interleukin (IL)-1β, IL-10, IL-6, IL-8, IL-2, monocyte chemoattractant protein (MCP)-1 and macrophage Inflammatory Protein (MIP)-1α among others (18–21). Cytokine levels vary across studies, even when the same testing platform has been used, possibly due to variations in the timing of sample collection, the synergistic interplay between pro- and anti-inflammatory cytokines, and variability in the strain of *Mtb* (19). Initial concentrations of pro-inflammatory cytokines like TNF-α and IFN-γ are highest on hospital admission followed by a subsequent decline over several weeks. Levels of intrathecal anti-inflammatory cytokines, such as IL-10, may be low if CSF samples are obtained when the inflammatory cascade is still developing (22–25). The ubiquitous finding across all studies is that CSF cytokine levels are elevated in TBM with some decrease after the initiation of treatment and inflammation continues despite drug administration. The degree of the attenuating influence of treatment, however, varies between cytokines. Combinations of inflammatory biomarkers could thus add value to the diagnosis of TBM. Numerous studies in pediatric TBM have examined the diagnostic accuracy of various combinations of host protein biosignatures in both serum and CSF taken on hospital admission. Protein combinations for CSF that have shown promising area under the curve (AUC), sensitivity and specificity include vascular endothelial growth factor (VEGF), myeloperoxidase (MPO) and IFN-γ (AUC = 0.97, sensitivity = 91.3, specificity = 100), as well as the combination of soluble intracellular adhesion molecule (sICAM)-1, MPO, CXCL-8 and IFN-γ (AUC = 0.97, sensitivity = 87, specificity = 95.8) (26). In serum a modified 7-protein biosignature developed for pulmonary TB [c-reactive protein (CRP), neural cell adhesion molecule (NCAM)-1, IFN-γ, CFH, apolipoprotein (Apo)-AI, IP-10 and serum amyloid A (SAA)] only showed modest sensitivity and specificity for pediatric TBM, but a 3 -protein signature (adipsin, Aβ42 and IL-10) was associated with improved diagnostic performance (27). While the potential of developing a bedside diagnostic tool for multiplexed proteins is intriguing, study sample sizes remain small and further validation is required in larger studies across the age range.

The association between CSF inflammatory mediators and various indicators of injury severity and outcome has yielded conflicting results. Several studies (19, 21, 22, 24, 28, 29) have found no association between the British Medical Research Council TBM stage (30) and the levels of TNF-α, IL-10, IL-1-β, IL-6 or IL-8. However, other studies show a significant positive correlation between the levels of TNF-α, IL-1β and IFN-γ and TBM stage (31, 32). Similarly, the association between CSF inflammatory biomarkers and outcome also is poor (20, 21, 24). Cumulatively these results indicate that while the cerebral inflammatory response is an important early disease process, biomarkers of inflammation do not necessarily reflect the degree of cerebral tissue injury and the severity of the disease; therefore, biomarkers of brain tissue injury may be important additional tools to predict and monitor disease severity.

### Brain-Specific Biomarkers

Brain-specific proteins have become valuable tools for diagnosis and prognostication in other forms of brain injury and infection, such as traumatic brain injury or stroke (33, 34). The cell-specificity may indicate the nature of cellular injury, their concentrations reflect injury severity, and their temporal profile provides insight into recovery or evolving injury (33). Only recently, brain-specific injury biomarkers have been investigated in TBM. A pediatric TBM study found elevated concentrations of CSF brain biomarkers S100B, glial fibrillary acidic protein (GFAP) and neuron specific enolase (NSE) which were associated with infarcts on brain imaging (20). Further, in serial samples over the first 4 weeks of hospitalization inflammatory mediator concentrations decreased in all patients, whereas these brain biomarkers continued to rise in patients who died and their trend over time was a promising prognostic biomarker (20). Similar findings have been reported in adult TBM (35) and a follow-up pediatric TBM study using whole genome transcriptomics in CSF confirmed the upregulation of genes and pathways associated with brain injury, including neuroexcitotoxicity (36). These studies highlight that injury processes initiated by the host inflammatory response are ongoing despite treatment. Further investigation into these mechanisms of injury is crucial to elucidate novel therapies directed at ameliorating brain injury, and brain-derived biomarkers will be an important tool in this quest.

### Compartmental Differences in Biomarker Concentrations

Adult and pediatric TBM data indicating that CSF cytokine concentrations are significantly greater than those seen in serum (20, 25) suggest compartmentalisation of the immune response at the site of disease, and a confounding effect of peripheral organs to serum cytokine levels. The detection of brain-derived biomarkers in blood is challenging and may additionally be influenced by their intrathecal concentration, their molecular weight and half-life (37). Brain-derived proteins can diffuse into the blood regardless of BBB breakdown (37, 38), but this is likely augmented when the BBB is compromised (39). Consequently, serum concentrations reflect only a fraction of CSF levels and only transiently. Although serum brain-specific injury biomarkers (such as S100B, GFAP and NSE) work well as diagnostic and prognostic tools in traumatic brain injury, they have been challenging to detect in TBM (20). This could be due to the extent of tissue injury, or the uncertainty around the timing of blood sampling relative to the onset of brain injury, which in TBM is likely to result from lasting injury processes rather than an acute discrete event. However, testing platforms used for TBM studies to date may have lacked adequate sensitivity to detect low quantity brain injury markers in blood. Newly developed assays with improved sensitivity (34) may warrant re-evaluating the role of serum-based brain biomarkers, especially as CSF requires invasive sampling.

Cerebrospinal fluid reflects changes in the brain more robustly than serum, implying that there is compartmentalisation within the CNS. Ventricular CSF, sampled as part of the management of TBM associated hydrocephalus, demonstrates significantly higher brain injury biomarker concentrations than lumbar CSF, while inflammatory biomarkers are greater in the lumbar compartment (20). This is similarly reflected in transcriptomic data, which showed upregulation of pathways associated with brain injury in the ventricular CSF and those associated with inflammation in the lumbar CSF (36). This likely reflects a decrement in brain-derived proteins along the brain-spine axis (37, 40) and the contribution of spinal sub-arachnoid inflammation present in as many as 76% of TBM patients (16). These data suggest that biomarker diagnostic, treatment, and prognostic thresholds must take the CSF compartment source into account.

## (Neuro)Inflammatory Markers in Pediatric HIV

### Pathogenesis of Pediatric HIV

Infection with HIV can cause a range of brain disorders, of which neurocognitive impairments is the most common phenomenon. HIV infects the CNS *via* transmigration of infected CD4^+^ cells and monocytes across the BBB (41, 42). Microglial cells and perivascular macrophages are cell types that subsequently become the source of chronic infection in the CNS (43). The pathogenesis of HIV-associated neurodevelopmental impairments in children is not fully understood. An aberrant immune regulation, characterized by chronic low-grade neuroinflammation is accepted to be a key mechanism that contributes to impaired brain functioning in children (44, 45) and adults (46) living with HIV. Viral proteins (e.g. Tat and gp120) that are released from infected cells activate microglial cells and astrocytes to produce pro-inflammatory cytokines and chemokines that impair neuron functioning when exposed over a chronic period.

### HIV Exposed Uninfected Children

World-wide and specifically in sub-Saharan Africa, important progress has been made in reducing vertical transmission of HIV to infants through the implementation of effective and widespread prevention of mother-to-child transmission (PMTCT) programmes (47, 48). While PMTCT success has resulted in the decline in pediatric HIV infection, discussed below, the number of HEU infants, i.e. perinatally exposed but not infected children, has rapidly risen. In 2018, the global population of HEU children was estimated to be 14.8, 13.2 million of whom resided in sub-Saharan Africa (49). Maternal HIV infection during pregnancy may have negative consequences for the development of the HEU child. Although HIV uninfected, the large population of HEU children is at increased risk of morbidity and mortality in general (50–52). Moreover, HEU children are at risk of impaired behavioral and neurocognitive functioning (53, 54). The prevalence of cognitive delay between 1 % and 31% and severe motor delay from 0 to 39% in HEU children was reported in a meta-analysis (55). A recent neuroimaging study showed that cortical surface area and thickness within frontal regions were associated with cognitive development, and in temporal and frontal regions with language development in HEU children (56). Impaired educational performance in HEU children (57) is a growing concern since these children may fail to progress academically and to acquire appropriate skills to sustain employment as adults in low- and middle-income countries (LMICs).

Our understanding of the pathogenesis of neurodevelopmental deficits in HEU children remains limited. This is in part due to the lack of appropriate animal models or post-mortem brain tissues from HEU children for neurobiological research. Evaluations of human systemic immune markers have provided important insights on the involvement of aberrant (neuro)immune regulation on neurocognitive delays in HEU children. In a South African birth cohort, increased granulocyte-macrophage colony-stimulating factor (GM-CSF), IFN-γ, IL-10, IL-12p70, IL-1β, IL-2, IL-4, IL-6 and neutrophil gelatinase-associated lipocalin (NGAL) in HEU infants, predicted worse motor functioning at 2-years follow-up (58). Interestingly, in this study maternal HIV infection was associated with lower levels of inflammatory markers in mothers and their children (e.g. IL-1β, IL-2, IL-4 and IFN-γ) compared to HIV uninfected mothers and their children (58), suggesting a suppressed immune profile in HEU children in this South African cohort. Similarly, another study with a Zimbabwean cohort also found decreased IL-6 levels in HEU children compared to HIV unexposed children (59). On the other hand, contradictory findings in European and American populations were reported. Increased circulating levels of IL-8 and IL-1β were detected in HEU infants as compared to unexposed infants in the Netherlands (60). Significantly increased levels of plasma IL-4 were found in Brazilian HEU children aged 6 to 12 years (61). Further, in Brazilian HEU neonates, increased circulating levels of IFN-γ and TNF-α compared to HIV unexposed neonates were reported (62). The conflicting findings between continents may be attributed to differences in HIV subtypes. HIV subtype Clade B is predominantly present in America, Western Europe, Australia and Asia and represents about 12% of the world's HIV infected population (63) whereas HIV subtype Clade C is present in countries of Southern Africa and India (64). Clade C tends to exert immunosuppressive effects as compared to the pro-inflammatory effects exerted by Clade B (52), which may explain the lower levels of inflammatory biomarkers reported in the Southern African cohorts. These studies underscore the importance of research on the involvement of the (neuro)immune system in neurodevelopmental delays in various African populations such as in Southern Africa, considering the expanding numbers of HEU children of mothers with predominantly HIV subtype Clade C, which represents about 50% of all HIV infections (64).

### Perinatally HIV Infected Children

Despite successful PMTCT programmes, millions of children are still born with HIV today (49). Children born with HIV (perinatally HIV, PHIV) show neurocognitive impairments as compared to uninfected peers, despite long term HIV suppression by combination antiretroviral therapy (cART). Studies reported a prevalence of severe cognitive delay between 21% to 35% and severe motor delays ranging from 14 to 81% in perinatally HIV infected children (55). The cause of these poorer neurocognitive outcomes as compared to peers is not well defined, but alterations in cerebral volume, white matter (WM) integrity, neurometabolites, and regional perfusion suggest underlying cerebral insults (65–68). HIV encephalopathy, a neurological disorder typical for children born with HIV is characterized by cerebral atrophy, basal ganglia calcifications, and white-matter abnormalities seen on conventional computed tomography or magnetic resonance imaging (MRI) (69). Even without these macrostructural imaging abnormalities, such as WM lesions (WML), microstructural WM injury as demonstrated by changes in diffusion values with diffusion-tensor imaging (DTI) is present in well treated PHIV-infected children (67, 69).

Long term HIV related immune activation may further contribute to this CNS pathology. HIV related systemic immune activation as indicated by systemic inflammation, monocyte and endothelial activation, with raised CRP, MCP-1, soluble CD14 (sCD14), soluble intercellular adhesion molecule-1 (sICAM-1) and vascular cell adhesion molecule-1 (sVCAM-1), IL-1, IL-6, IL-8, IL-10, IL-18, TNF-α), and soluble TNF receptor II (sTNF-RII) concentrations is reported in well treated PHIV (70–73). In HIV infected adults, elevated sCD14 levels in CSF were associated with increased levels of CSF neurofilament light-chain (NfL) levels and reduced brain tissue levels of the neurometabolite N-acetylaspartate (NAA) (74, 75).

In general, in children and more specifically in PHIV children, reports on intrathecal markers are scarce. In a recent Dutch cohort study, well treated PHIV children had increased systemic CRP, IFN-γ, IP-10, and MCP-1 as compared to controls, indicative of immune activation and inflammation. These children had suppressed HIV viral load levels in both blood and CSF (76). Intrathecal markers of immune activation and inflammation such as sCD14, and IL-6, and NFH were not elevated in CSF, but relative elevation of these markers within the normal range were associated with poorer cerebral and cognitive outcomes, indicating that immune activation and neurodegeneration may play a role in pediatric HIV related cerebral insults (76). In addition to associations of immune activation markers and neurodegeneration, an association between HIV related inflammation and neuroretinal thinning (as measured by Optical Coherence Tomography) in a cohort of cART treated perinatally HIV-infected children was detected (77). Ongoing immune activation, inflammation, and neuronal injury could therefore occur simultaneously with retinal thinning in PHIV. Taken together, one may postulate that chronic HIV related immune activation, inflammation and microstructural neuronal injury may precede functional neurocognitive impairments and macrostructural MRI abnormalities.

### TB and HIV Co-infection

People living with HIV are 18 times at risk to develop active TB disease as compared to HIV uninfected people (6). Interestingly, HEU children are also at significant risk at TB infection (78, 79). HIV and TB coinfection in children has become an important challenge to diagnose and manage globally. Moreover, TB and HIV coinfection potentially exacerbate each other's negative effects on the CNS. Evidence from a computed tomography imaging study showed that PHIV children with clinically diagnosed TBM presented with higher ventricular enlargement, gyral enhancement and cerebral atrophy as compared to HIV-negative children (80). Even though the effects of TB co-infection on cognitive functioning in PHIV or HEU children is unclear, a study in Zambian adults with HIV showed that co-infection with TB significantly contributed to impaired cognitive function as compared to people with HIV but without TB (81). It is therefore reasonable to hypothesize that TB and HIV co-infection in children will lead to poorer brain health and neurocognitive performance than these infections independently. The immune system may play a pivotal function in the potentiating effects between HIV and TB infections (82), and possibly their effects on the brain. For example, the proportion of peripheral blood CD14^+^CD16^+^ monocytes are higher in TB and HIV coinfected patients as compared to people living with HIV but without TB infection (83). CD14^+^CD16^+^ monocytes that are infected with HIV, migrate across the BBB, which is the primary mechanism by which HIV infects the CNS (84), resulting in cognitive impairment. Hence, TB infection may facilitate neurocognitive disorders in HIV patients by increasing the CD14^+^CD16^+^ monocyte subset. In human post-mortem brain tissues, it was found that patients with TB and HIV co-infection had increased markers of activated microglia and astrocytes in certain brain regions as compared to patients that only had TB or HIV (85). Therefore, TB and HIV co-infection can have an additive effect on neuroinflammatory regulation, which is potentially reflected by peripheral blood (neuro)inflammatory markers. Literature on the associations of biomarkers of neuroinflammation and neuronal injury with impaired brain health in children with TB and HIV coinfection is lacking and an important topic for future studies.

## Discussion and Outlook

The presented literature suggests that TBM and HIV are associated with increased intrathecal immune responses, the temporal profile and extent of increase are likely dependent on the disease mechanisms. The pathogenesis of TBM and pediatric HIV differs. TBM represents a more acute infection while neuro-HIV follows a more chronic infective process. In both cases diagnosing these conditions and determining their impact on the brain is difficult. By discussing these two conditions this review hopes to offers insights into the generalizable use of biomarkers across the spectrum of CNS infection, those which are acute and often short-lived with treatment, as well as those which persist and manifest over the longer term. In addition to the classical increased pro-inflammatory cytokines, TBM is characterized by cytokine changes induced by acute neuronal and vascular damage, whereas pediatric HIV involves a chronic low-grade neuroinflammatory response to products of CNS HIV infection.

Given the relevance of early inflammatory increases in pathologies like TBM, blood-based inflammatory biomarkers are highly needed. However, in view of the current lack of brain-specific inflammatory biomarkers, this is a challenge. A possible solution could be the analysis of inflammatory mediators or their transcripts in brain-derived exosomes in plasma (86, 87), which could confer desirable brain-specificity in blood.

Given the dynamic character of the immune-response and the involvement of several immune-related markers, it is likely that profiles or arrays of different markers should be measured. Novel multiplexing technologies enable such profile analysis, and different platforms are available. While these technologies may differ in sensitivity of detection of low circulating levels of these inflammatory molecules in CSF, they also differ in costs of instrumentation, reagents and level of automation. It is expected that some of the more affordable technologies may even become available in bed-side point of care formats, which is especially relevant in LMICs. Once the wet-analysis is finalized, profile analysis requires statistical tools for interpretation, for which algorithms or Apps could be developed to enable interpretation for the individual patient.

To date, few studies have taken advantage of novel ultrasensitive technologies to measure brain-injury biomarkers in blood, especially Neurofilament Light (NfL). From the studies performed in TBM, it appears that brain injury markers may have prognostic value, which is now robustly being shown for NfL in other chronic and acute diseases, such as SARS-CoV2-related encephalitis (4, 88, 89). With reference to pediatric diseases, blood biomarkers NfL and GFAP are increased in children with acute demyelinating disorders and have potential value for the decision who to treat, and to monitor therapeutic responses (90). Interestingly, levels of these biomarkers are relatively high in healthy newborns and children, which may allow the use of less expensive technologies. For example, Beerepoot et al. showed that levels of blood based neurobiomarkers NfL and GFAP concentrations show a U shape across the lifespan: they are high in newborns, and the lowest levels probably are reached around age 15, after which they increase again (90).

Biomarker studies in any disease requires an infrastructure of biobanking and systematic recording of clinical and other relevant disease characteristics, in addition to sufficient funding to perform such studies. In addition, pre-analytical aspects may be another challenge. For some biomarkers, samples are ideally processed within a couple of hours after collection (91). Different processing solutions should thus be defined for specific biomarkers for use in a variety of settings. Fortunately, a stringent pre-analytical protocol is not required for some biomarkers, like NfL and GFAP (91).

In conclusion, there is clearly a strong need for and demonstrated value of fluid biomarkers to aid precise biological diagnosis of neuroinfectious disorders highly prevalent on the African continent. With the current technological developments in other disease areas, more technological opportunities become within reach to measure disease relevant proteins in accessible matrices. It is of utmost importance that these technologies are transformed into tools that can be implemented in resource-low conditions to enable access to these healthcare improvements in a globally equitable way that maximizes benefit to patients.

## Author Contributions

All authors drafted an equal part of the manuscript and all have critically revised the different versions of the manuscript until its final version.

## Funding

PN was supported by a Wellcome Trust Intermediate Fellowship (222020/Z/20/Z).

## Conflict of Interest

The authors declare that the research was conducted in the absence of any commercial or financial relationships that could be construed as a potential conflict of interest.

## Publisher's Note

All claims expressed in this article are solely those of the authors and do not necessarily represent those of their affiliated organizations, or those of the publisher, the editors and the reviewers. Any product that may be evaluated in this article, or claim that may be made by its manufacturer, is not guaranteed or endorsed by the publisher.
